# Combinational expression of tumor testis antigens NY-ESO-1, MAGE-A3, and MAGE-A4 predicts response to immunotherapy in mucosal melanoma patients

**DOI:** 10.1007/s00432-022-04514-z

**Published:** 2022-12-17

**Authors:** Sandra N. Freiberger, David Holzmann, Grégoire B. Morand, Martin Hüllner, Mitchell P. Levesque, Reinhard Dummer, Viktor H. Koelzer, Niels J. Rupp

**Affiliations:** 1 Department of Pathology and Molecular Pathology, University Hospital Zurich, Schmelzbergstrasse 12, 8091 Zurich, Switzerland; 2 Faculty of Medicine, University of Zurich, Zurich, Switzerland; 3 Department of Otorhinolaryngology - Head and Neck Surgery, University Hospital Zurich, Zurich, Switzerland; 4 Department of Otolaryngology - Head and Neck Surgery, Sir Mortimer B. Davis - Jewish General Hospital, McGill University, Montreal, QC Canada; 5 Department of Nuclear Medicine, University Hospital Zurich, Zurich, Switzerland; 6 Department of Dermatology, University Hospital Zurich, Zurich, Switzerland

**Keywords:** Immunotherapy, Biomarker, Cancer testis antigen, Immune response, Mucosal melanoma

## Abstract

**Purpose:**

Immunotherapy using immune checkpoint inhibitors (ICI) has revolutionized cancer treatment in recent years, particularly in melanoma. While response to immunotherapy is associated with high tumor mutational burden (TMB), PD-L1 expression, and microsatellite instability in several cancers, tumors lacking these biomarkers can still respond to this treatment. Especially, mucosal melanoma, commonly exhibiting low TMB compared to cutaneous melanoma, may respond to immunotherapy with immune checkpoint inhibitors. Therefore, the aim of our study was to investigate novel biomarkers in mucosal melanoma that predict response to combined ipilimumab and nivolumab.

**Methods:**

We investigated 10 tumor samples from 10 patients (three responders, seven non-responders) before treatment and six tumor samples from five patients after progression using a targeted Next Generation Sequencing (NGS) gene expression panel. The findings were corroborated with an independent method (i.e., immunohistochemical staining) on the same 10 tumor samples before treatment and, to increase the cohort, in addition on three tumor samples before treatment of more recent patients (one responder, two non-responders).

**Results:**

With the targeted gene expression panel, we found the three tumor testis antigens *CTAG1B* (*NY-ESO-1*), *MAGE-A3*, and *MAGE-A4* to be predominantly expressed in responding tumors. This marker panel was either not or not completely expressed in non-responders (*p* < 0.01). Using immunohistochemistry for all three markers, we could confirm the elevated expression in tumors responding to the ipilimumab/nivolumab combination therapy.

**Conclusion:**

In conclusion, these three biomarkers await validation in a larger patient cohort and could be easily used in future routine diagnostics to predict the outcome of ipilimumab/nivolumab combination therapy in mucosal melanoma patients.

**Supplementary Information:**

The online version contains supplementary material available at 10.1007/s00432-022-04514-z.

## Introduction

The advent of immune checkpoint inhibition had an immense impact on cancer treatment. Large clinical studies have shown the success of immunotherapy in a variety of cancers (Borghaei et al. 2015; Hellmann et al. 2019; Larkin et al. 2015), and numerous investigations were performed to identify predictive biomarkers for treatment response. A high tumor mutational burden (TMB) (Yarchoan et al. 2017) and strong expression of programmed death ligand 1 (PD-L1) (Topalian et al. 2012) were shown to be broadly associated with response to immunotherapy in several cancer types and were suggested as tumor-agnostic biomarkers. These results led to the approval of specific immune checkpoint inhibitors (ICI) in certain cancer types, depending on the positivity of certain biomarkers (e.g., PD-L1 positivity in non-small cell lung cancer (NSCLC) for pembrolizumab treatment (Pembrolizumab (Keytruda) for first-line treatment of metastatic NSCLC. 2017), microsatellite-instability-high (MSI-H), or mismatch-repair-deficient (dMMR) solid tumors (In brief: Pembrolizumab (Keytruda) for cancers with biomarkers. 2018)).

In mucosal as well as cutaneous melanoma, however, patients with low TMB or negative PD-L1 staining could still be treatment responders (Carlino et al. 2018; Samstein et al. 2019). Immunotherapy is thus approved both in the adjuvant and advanced setting, irrespectively of specific biomarkers (Nivolumab (Opdivo) plus ipilimumab (Yervoy) for metastatic melanoma. 2015). Moreover, ICI in the neoadjuvant setting may play a larger role in the future (Lee and Brady 2021).

Studies on mucosal melanoma and ICI are rare; however, a subset of mucosal melanoma patients responds to ICI treatment (23.3.-37.1%) (D’Angelo et al. 2017). As side effects can be severe, specific biomarkers to predict the response to immunotherapy in this patient group are desired. It was shown that 91% of melanoma patients treated with a combination of ipilimumab and nivolumab had side effects, with 54% of patients suffering from grade 3 or 4 side effects (Postow et al. 2015). Since current biomarkers are inconsistently associated with response in melanoma, patients today have to accept such a high risk of side effects.

Commonly, mucosal melanoma has low TMB (Freiberger et al. 2019; Hayward et al. 2017), making it a non-suitable marker. Moreover, a single marker may not be sufficient to predict ICI response, as shown in the Checkmate 026 study for NSCLC, where patients with a combination of high TMB and strong PD-L1 positivity showed longer progression-free survival (PFS) than patients with positivity of either marker (Peters et al. 2017). To perform an in-depth analysis for possible biomarkers, or biomarker combinations for response to ICI, we performed differential gene expression analysis, using a targeted next-generation sequencing (NGS) assay including 395 genes (Oncomine Immune Response Assay, OIRA) designed for the quantification of immune cell and inflammatory transcripts. We then confirmed our results by immunohistochemistry on a well-characterized subset of mucosal melanoma cases with full clinicopathological data and response information.

## Materials and methods

### Patient material/ethics statement

Surplus tumor material from formalin-fixed paraffin-embedded (FFPE) specimens was used for this study (see Table 1 for details). For the predictive biomarker approach, we investigated tumor samples before ipilimumab/nivolumab treatment from ten patients (three responders, seven non-responders). To investigate the evolution of marker expression during therapy, we added six tumor samples after progression from five of the seven non-responders for analysis. Ethical approval was given by the cantonal ethics commission (BASEC 2020–01663, approved: 30 July 2020) and all patients signed written informed consent (BASEC PB_2017-00,494, amendment approved: 25 July 2017). All patient material for this study was reviewed by an experienced attending pathologist (NJR). Two patient cases (SIT3 and 4) have been published previously (Freiberger et al. 2021).

**Table 1:** Patient cohort

Sample ID	Gender	Primary location	Material before ICI	Mutational load n (mut/Mb)	Interval between tumor excision and start of ICI (months)	Time point of response assessment after start of ICI (months)	Response to ICI	Performed analysis
SIT1	Male	Sinonasal	Primary tumor	8 (12.3)	4.5	11.3	No	OIRA/IHC
SIT2	Female	Sinonasal	Local recurrence	6 (9.2)	1.2	27.4	Yes	OIRA/IHC
SIT3	Male	Sinonasal	Metastasis	22 (34.9)	2.5	4.7	No	OIRA/IHC
SIT4	Male	Sinonasal	Primary tumor	6 (9.2)	8.2	25.2	No	OIRA/IHC
SIT6	Female	Sinonasal	Primary tumor	2 (3.1)	1.1	43.4	Yes	OIRA/IHC
SIT7	Male	Sinonasal	Primary tumor	NA	15.2	30.7	Yes	OIRA/IHC
SIT13	Male	Anal	Primary tumor	13 (20.0)	4.9	1.4	No	OIRA/IHC
SIT15	Female	Vaginal	Metastasis	NA	19.4	6.1	No	OIRA/IHC
SIT18	Male	Sinonasal	Primary tumor	13 (20.0)	75.4	15.6	No*	OIRA/IHC
SIT19	Female	Vaginal	Primary tumor	10 (15.9)	1.2	3.7	No	OIRA/IHC
SIT20	Female	Sinonasal	Local recurrence	NA	1.3	2.4	Yes	IHC only
SIT21	Female	Sinonasal	Local recurrence	NA	2.0	5.0	No	IHC only
SIT22	Female	Sinonasal	Primary Tumor	NA	4.2	1.2	No	IHC only
Samples *n* = 13	Male *n* = 6 Female *n* = 7	Sinonasal *n* = 10 Anal *n* = 1 Vaginal *n* = 2	Primary *n* = 8 Recurrence *n* = 3 Metastasis *n* = 2				Yes *n* = 4 No *n* = 9	
Age at diagnosis (median (range) years): 71 (51–86)								

### Evaluation of response to ipilimumab/nivolumab therapy

All patients received regular FDG-PET/CT scans during the therapy and follow-up period. FDG-PET/CT evaluation was performed according to the PET Response Evaluation Criteria for Immunotherapy (PERCIMT).

### RNA isolation

RNA from FFPE specimens was isolated by automated extraction, using the Maxwell^®^ 16 LEV RNA FFPE Purification Kit (Promega, Madison, WI, USA) according to the user manual. Quantification of RNA was performed using a fluorometric assay (Qubit, Thermo Fisher Scientific, Waltham, MA, USA).

### Library preparation and sequencing

Library preparation for the Oncomine™ Immune response assay (OIRA, Thermo Fisher Scientific) was performed according to the manufacturers’ protocol, using 10 ng of input RNA. Libraries were pooled and diluted to 50 pM and then templated and loaded on 530 or 540 chips using the Ion Chef™ instrument (Thermo Fisher Scientific). Sequencing was conducted on the Ion S5™ sequencer (Thermo Fisher Scientific).

### Sequencing data analysis

Sequencing data were analyzed using the Affymetrix™ Transcriptome Analysis Console (Affymetrix, Santa Clara, CA, USA). To assess differential gene expression, samples were assigned to different groups (responders and non-responders, or responders, non-responders before treatment, non-responders at progression). To avoid a batch effect, batch correction was taken into account, using the respective feature of the Affymetrix™ Transcriptome Analysis Console.

### Immunohistochemistry

For immunohistochemistry 2 µm sections were cut from the same blocks as for the Immune Response Assay. In addition, three more patient samples were analyzed with IHC only (see Table 1). Immunohistochemical staining with the monoclonal mouse anti-human NY-ESO-1 antibody clone E978 (Thermo Fisher Scientific) was performed on the Bond III automated staining system in a 1:10 dilution (Leica, Wetzlar, Germany). The optiView DAB-kit was used for detection. The IHC-plus™ MAGE-A3 monoclonal antibody clone 4E1 and the IHC-plus™ MAGE-A4 monoclonal antibody clone 1F9 (both Lifespan Biosciences, Seattle, WA, USA) were used in a 1:100 dilution and staining was performed on the Ventana Benchmark automated staining system (Ventana, Oro Valley, AZ, USA). All stained slides were evaluated by an experienced senior attending pathologist (NJR) and analyzed in a semi-quantitative manner (scoring: 0 = negative, 1 = weak, 2 = intermediate, 3 = strong). In heterogeneous cases, the two predominant patterns were evaluated and the mean was calculated.

### Statistical analysis

Median and range (min–max) are given for descriptive analysis of continuous variables. Semi-quantitative immunohistochemistry data were analyzed using the Mann–Whitney *U *Test. Binary variables were associated in contingency tables using the two-sided Fisher’s exact test. Survival curves were built according to the Kaplan–Meier method and the log-rank test was used to compare factors. Statistical analyses were performed using SPSS^®^ 27.0.0.0 software (IBM^©^, Armonk, NY, USA). A *p* value of < 0.05 was considered to indicate statistical significance.

## Results

We initially identified ten mucosal melanoma patients treated with combinational ipilimumab/nivolumab immunotherapy in our hospital between January 2017 and March 2022. (Table 1, SIT1-SIT19). Three patients responded to the treatment, while seven patients did not respond. One of the non-responders (SIT18) showed an initial response, but progressed eventually. Response to ICI treatment was independent of the mutational burden of the tumor before therapy (Table 1).

**Fig. 1:**
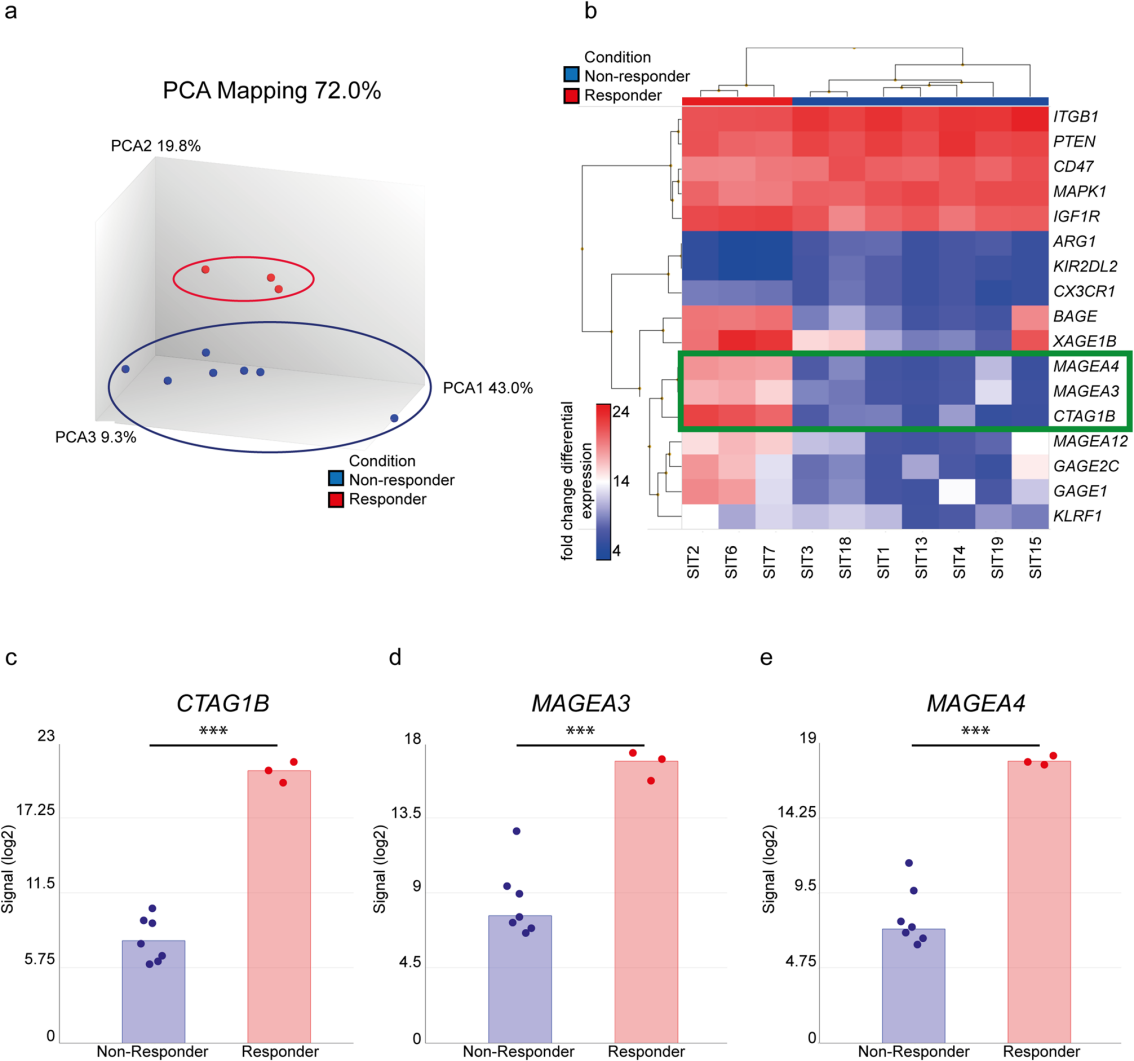
RNA expression analysis. **a** Principal component analysis (PCA) of responders (red) and non-responders (blue). **b** Heat map of genes significantly altered between responders (red) and non-responders (blue). The color legend displays fold change differential expression. **c** Gene expression of *CTAG1B* (*NY-ESO-1*). **d** Gene expression of *MAGEA3*. **e** Gene expression of *MAGEA4*.. ***p < 0.001

To identify possible biomarkers that predict the response to combined ipilimumab/nivolumab, an RNA expression panel (Oncomine Immune Response assay, OIRA) was used. A total of ten mucosal melanomas, three from responders and seven from non-responders, were analyzed using the Affymetrix™ Transcriptome Analysis Console. Principal component analysis (PCA) separated responders from non-responders (Fig. 1a). Analysis of differential gene expression indicated different clusters of responders and non-responders (Fig. 1b). Six genes were upregulated and 11 genes were downregulated. *MAGE-A3*, *CTAG1B* (*NY-ESO-1*) and *MAGE-A4* appeared to be the genes with the highest significance (*p* < 0.001) and by far lowest false discovery rate (*q* < 0.01) (Fig. 1c,d,e; supplementary fig. S1). Other genes with significant differential expression had a high FDR and were therefore not considered (supplementary fig. S1). We did not detect any remarkable differences in the expression of genes involved in lymphocyte regulation, cytokine signaling, or immune checkpoints.

To investigate marker evolution after ICI, we included metastases emerging during ICI therapy of progressing patients, as well (Table 2). Five non-responders had tumor material after ICI treatment available and were analyzed.

**Table 2:** Sample cohort of non-responders for marker evolution analysis

Sample ID	Primary location	Material before ICI	Material at progression	Time from ICI start to progression (months)
SIT1	Sinonasal	Primary tumor	Skin metastasis	11.3
SIT3	Sinonasal	Metastasis lymph-node level IV	Tongue metastasis	4.7
SIT4	Sinonasal	Primary tumor	1. Local recurrence 2. Local recurrence	25.2 31.2
SIT13	Anal	Primary tumor	Metastasis lymph node level I	1.4
SIT19	Vaginal	Primary tumor	Lung metastasis	3.7

PCA and expression analysis showed no clear separation of non-responding tumors before and after ICI therapy (Supplementary Fig. 2a and 2b). Expression of *CTAG1B* (*NY-ESO-1*) and *MAGE-A4* showed no significant difference between pre- and post-ICI tumors. Expression of *MAGE-A3* was even less in post-ICI tumors compared to pre-ICI tumors (supplementary Fig. 2c, 2d, 2e).

**Fig. 2:**
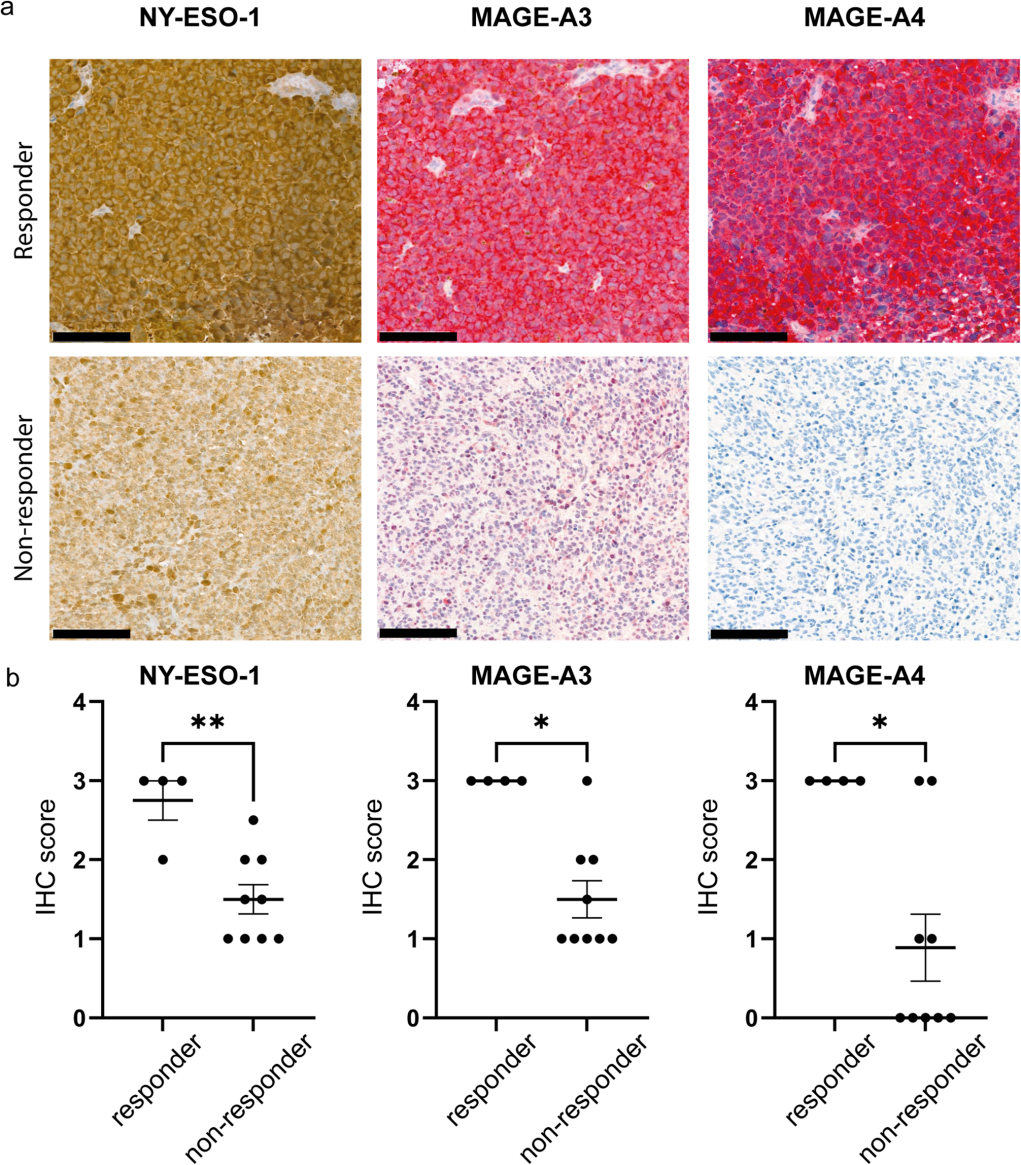
Protein level analysis. **a** Immunohistochemical staining of NY-ESO-1, MAGE-A3, and MAGE-A4, showing representative images. **b** Corresponding semi-quantitative immunohistochemical expression analysis of NY-ESO-1, MAGE-A3, and MAGE-A4 Scale bar: 200 um. Graphs show mean ± SEM. *p < 0.05; **p < 0.01

To investigate whether the expression of these three cancer testis antigens is translated to protein level, immunohistochemistry was performed on tumors before ipilimumab/nivolumab treatment for the three corresponding proteins MAGE-A3, MAGE-A4, and NY-ESO-1 (Fig. 2a). The staining was performed on the same FFPE blocks as the Immune Response Assay. In addition, we extended the cohort with three more recent patient samples before ipilimumab/nivolumab treatment (until October 2022; Table 1 SIT20-SIT22). In total, 13 patient samples were analyzed (see Table 1). Analysis of the staining was done semi-quantitatively. In all cases, the staining of MAGE-A3 and MAGE-A4 was cytoplasmic, while for NY-ESO-1, it was predominantly cytoplasmic with few nuclear signals. All tumors of the responders showed a mainly homogenous staining for all three markers with a positivity in > 90% of the tumor area. Tumors of non-responders showed a higher level of heterogeneity.

Immunohistochemical expression of NY-ESO-1, MAGE-A3, and MAGE-A4 was significantly higher in responding melanomas (*p* = 0.007, *p* = 0.014, *p* = 0.028 (Fig. 2b). In all cases, responding tumors showed a positivity (score 2 or 3) of all three markers with a homogeneous expression pattern. All resistant tumors showed a weak and/or incomplete expression of the 3-marker immunohistochemistry panel. Moreover, the expression pattern was more heterogeneous.

**Fig. 3:**
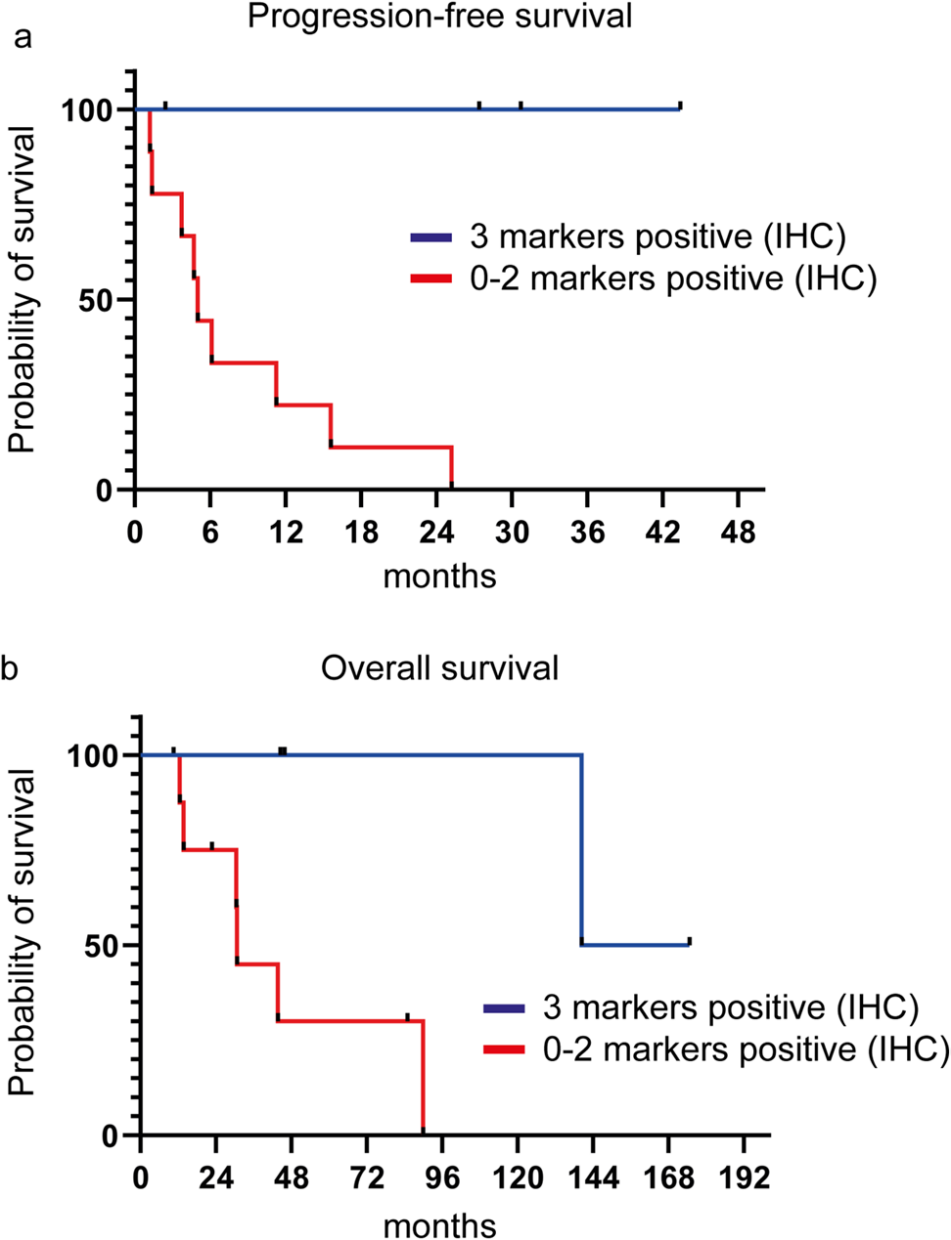
Survival curves of patients with immunohistochemical positivity of all three markers and patients with immunohistochemical positivity of less than three markers. **a** Progression-free survival (*p* = 0.006). **b** Overall survival (*p* = 0.016); IHC immunohistochemistry

Statistical analysis by Fisher’s exact test indicated that the positivity of all three markers is associated with response to ipilimumab/nivolumab therapy (*p* = 0.001). Progression-free and disease-specific survival were significantly decreased in patients with tumors expressing none to two markers, while patients with tumors expressing MAGE-A3, MAGE-A4, and NY-ESO-1 altogether show ongoing responses (Fig. 3a,b; Log-rank test: *p* = 0.006/*p* = 0.016). When considering each marker individually, their expression correlates with disease-specific survival, while PFS only correlates with expression of NY-ESO-1 or MAGE-A3 (supplementary Fig. 3).

## Discussion

In our exploratory study, we aimed to identify suitable biomarkers for the response to ipilimumab/nivolumab combination immunotherapy in mucosal melanoma patients. Our final cohort consisted of 30.8% responders (four out of 13 patients). This is in concordance with previous studies, reporting a response rate of 23.3–37.1% (D’Angelo et al. 2017). TMB, a potential biomarker for response to immunotherapy in other cancer types (Samstein et al. 2019), plays a only minor role in melanoma, as these patients may respond independently of TMB. The CheckMate067 trial resulted in an overall response rate (ORR) of 64.8% for patients with a high mutational burden and an ORR of 51.0% for patients with a low mutational burden (Hodi et al. 2021). Likewise, our data support this independency, as the mutational load of our responders was even lower than the mutational load of the non-responders (Table 1).

Using a RNA-based 395-gene expression panel on tumors before combinational ipilimumab/nivolumab immunotherapy, we did not detect any remarkable differences in the expression of genes involved in lymphocyte regulation, cytokine signaling, or immune checkpoints. Moreover, visual inspection of CD8 immunohistochemistry did not reveal differences in CD8^+^ T-cell infiltration (data not shown). Although some of these genes were recently associated with immune checkpoint inhibition, we decided to focus on genes that were highly differentially expressed and had the lowest *p* value and FDR. A significant difference in the expression of the three cancer testis antigens *NY-ESO-1*, *MAGE-A3*, and *MAGE-A4* was evident and confirmed on protein level by immunohistochemistry in a slightly increased cohort. Cancer testis antigens were previously shown to be expressed on either embryonic tissue or on several types of tumor cells, including melanoma, while expression on normal tissue is not evident (Jungbluth et al. 2001). Moreover, expression of NY-ESO-1 was associated with reduced relapse-free survival (Svobodová et al. 2011). Of the three detected cancer testis antigens, especially NY-ESO-1 was shown to induce a humoral immune response, as antibodies against the protein were detected in cancer patients (Oshima et al. 2016). Further, a cellular immune response is elicited in terms of NY-ESO-1-specific CD8 + T cells in melanoma patients (Jäger et al. 2000). Due to the immunogenicity of NY-ESO-1, it was used as a target in vaccination trials, studies with adoptive T-cell transfer and in combination with immunotherapy to boost immune response against the NY-ESO-1 expressing cancer cells (Thomas et al. 2018). Moreover, CTLA-4 blockade by ipilimumab enhanced NY-ESO-1 antigen-specific B-cell and T-cell immune responses in patients with durable objective clinical responses and stable disease (Yuan et al. 2008). This is because blocking CTLA4 allows proliferation of tumor-specific *T* cells, which are most probably, also directed against other cancer testis antigens. A recent study on unresectable or metastatic melanoma patients using an NY-ESO-1 vaccine plus ipilimumab showed stable disease as best clinical response. This was associated with the presence of specific antibodies and *T* cells against NY-ESO-1 (Slingluff et al. 2021). Fässler et al. investigated the presence of antibodies against melanocyte differentiation antigens and cancer testis antigens in serum samples from stage IV melanoma patients before immunotherapy. Responders showed a higher level of antibodies against NY-ESO-1, Melan A, TYRP1, and TYRP2, concluding that these were suitable candidates to predict immunotherapy outcome (Fässler et al. 2019).

Altogether, these studies indicate that blocking immune checkpoints by immunotherapy and resulting cellular immune responses directed against cancer testis antigens are an efficient combination to eliminate the tumor. While we did not see differences in CD8 T-cell infiltration and, in PD-L1 expression (data not shown), CD8 T-cell infiltration is generally lower in mucosal compared to cutaneous melanoma (Nakamura et al. 2020). Therefore, ICI alone might not be sufficient to elicit an anti-tumor response. Thus, an additional trigger for the immune system, possibly via cancer testis antigen expression, is needed, and would allow a synergistic anti-tumor response by T cells and tumor-directed antibodies. This is in concordance with our finding of mucosal melanomas expressing cancer testis antigens, showing a clear advantage in response to immunotherapy. Moreover, the expression of these antigens was homogeneously distributed in responding tumors, while the expression was either low and/or heterogeneously distributed in tumors of non-responders. Therefore, in tumors with heterogeneous expression, the weaker expressing areas may contribute to therapy resistance. This is also supported by our NGS analysis regarding marker evolution. All three markers are either equally low or even lower expressed in post-ICI samples. No significant changes in morphology were found in the samples before and after immunotherapy (data not shown); however, in two cases, an additional spindle cell differentiation was evident as published recently (Freiberger et al. 2021). This is concordance with our previously published data that molecular genotype shows no correlation with morphology in sinonasal melanoma (Freiberger et al. 2019). However, our study is limited by the lack of patient blood samples, so that the detection of antibodies against cancer testis antigens is not possible.

Current clinical trials study the effect of cancer testis antigen vaccination in combination with immunotherapy. One trial examines the combination of NY-ESO-1 and pembrolizumab in ovarian cancer, NSCLC, esophageal squamous cell carcinoma, and other solid tumors (NCT04939701). Another one studies the combination of NY-ESO-1 and MAGE-A3 with standard of care treatment, which includes immunotherapy (NCT04908111) in NSCLC. Whether outcomes will be significant and side effects will be acceptable remains unsettled.

In conclusion, although the sample size of our cohort is limited, owing to the rarity of the investigated tumor entity, we found that the combined expression of all three markers was strongly associated with response to ipilimumab/nivolumab therapy in mucosal melanoma patients. Further validation in larger patient cohorts will be needed. However, immunohistochemical staining of melanoma resection specimen is simple and quick to perform and can easily be implemented into the routine diagnostic setting. This three-biomarker-combination test could prevent non-responders from a treatment with possible severe side effects and supplies predicted responders with suitable treatment.

## Supplementary Information

Below is the link to the electronic supplementary material.

Supplementary file1 (TIF 8806 KB)

Supplementary file2 (TIF 25526 KB)

## Data Availability

The datasets generated during and/or analyzed during the current study are available from the corresponding author on reasonable request.
