# Supplementary figures and images for: Combinational expression of tumor testis antigens NY-ESO-1, MAGE-A3, and MAGE-A4 predicts response to immunotherapy in mucosal melanoma patients
Source: J Cancer Res Clin Oncol. 2022 Dec 17;149(9):5645–53. doi: 10.1007/s00432-022-04514-z (PMC10356647; doi:10.1007/s00432-022-04514-z)

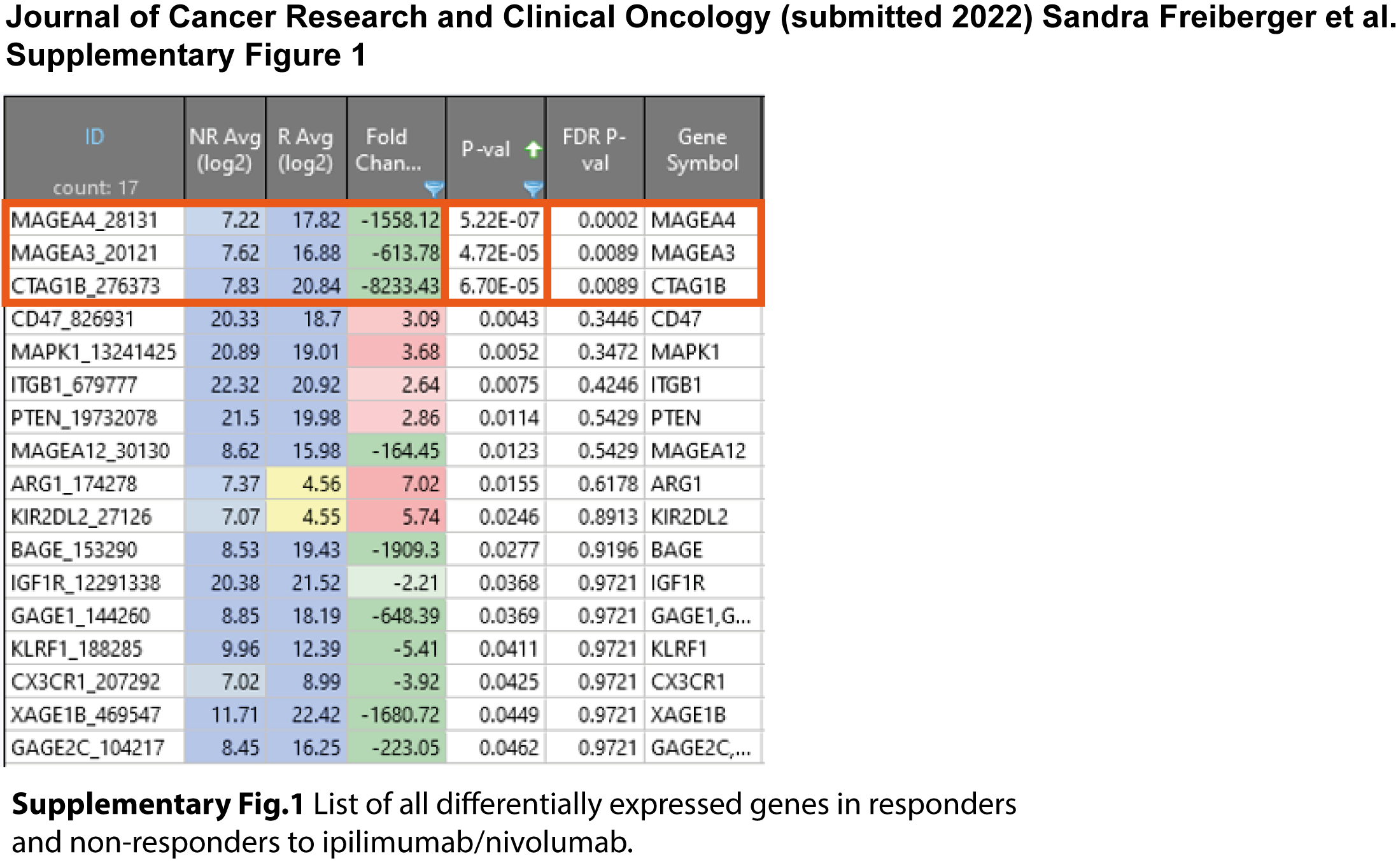

Supplement: Supplementary file 1 — Supplementary file1 (TIF 8806 KB) [file 432_2022_4514_MOESM1_ESM.tif]

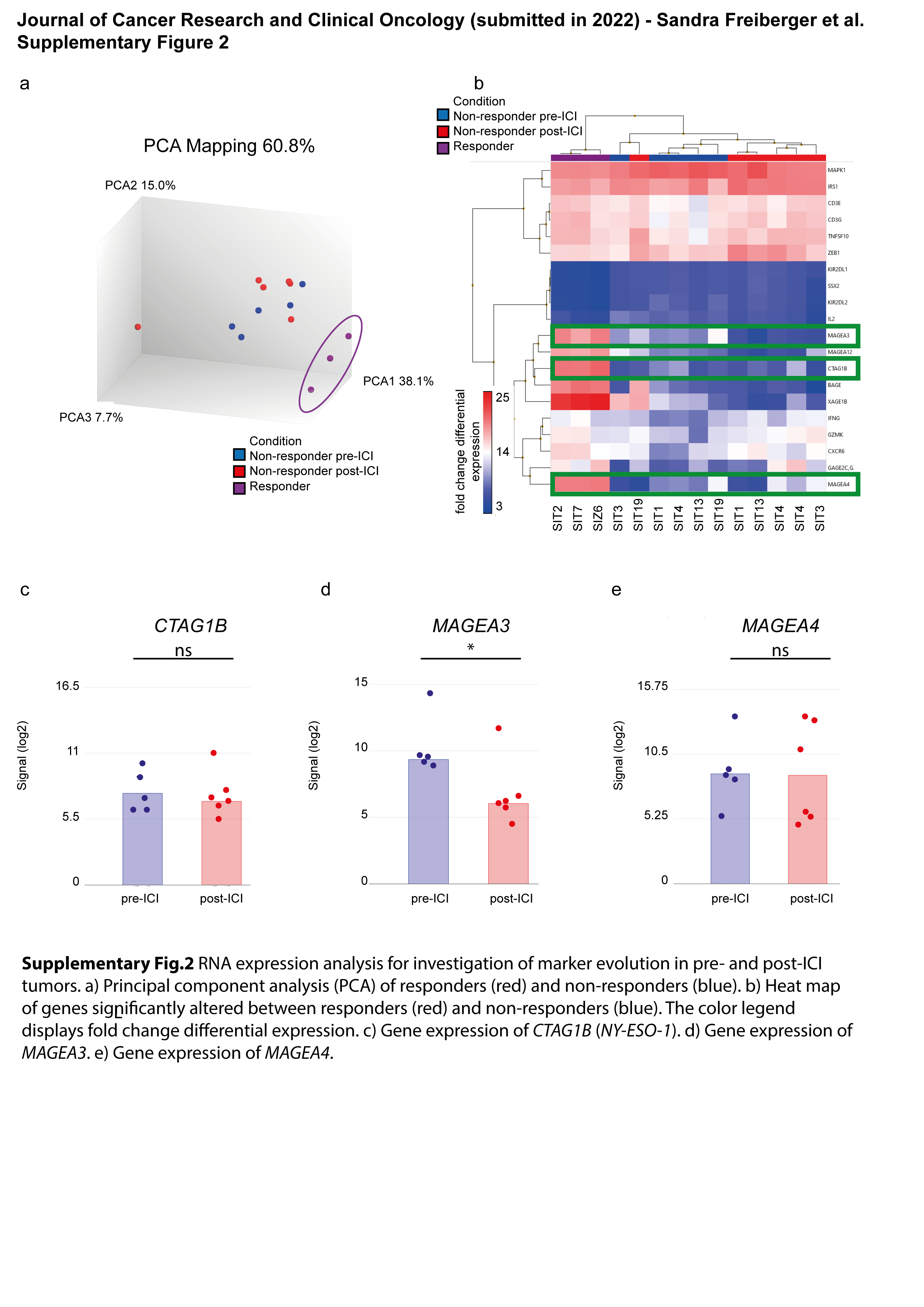

Supplement: Supplementary file 2 — Supplementary file2 (TIF 25526 KB) [file 432_2022_4514_MOESM2_ESM.tif]
